# Parental manipulation of offspring size in social groups: a test using paper wasps

**DOI:** 10.1007/s00265-019-2646-3

**Published:** 2019-02-25

**Authors:** Christelle Couchoux, Jeremy Field

**Affiliations:** Centre for Ecology & Conservation, University of Exeter, Penryn campus, Penryn, Cornwall, TR10 9FE UK

**Keywords:** Aggression, Body size, Eusociality, Foraging behavior, Parental manipulation, *Polistes*

## Abstract

**Abstract:**

Maternal effects should be especially likely when mothers actively provision offspring with resources that influence offspring phenotype. In cooperatively breeding and eusocial taxa, there is potential for parents to strategically manipulate offspring phenotype in their own interests. Social insect queens are nearly always larger than their worker offspring, and queens could benefit by producing small daughter workers in several ways. If queens use aggression to dominate or coerce workers, a queen producing small workers might minimize potential conflict or competition from her offspring. In addition, because of the trade-off between the number of workers she is able to produce and their individual size, a queen may produce small workers to optimize colony work effort. In this study, we investigate why queens of the primitively eusocial paper wasp *Polistes gallicus* limit the size of their workers. We created queen–worker size mismatches by cross-fostering queens between nests. We then tested whether the queen–worker size difference affects worker foraging and reproductive effort, or the amount of aggression in the group. Some of our results were consistent with the idea that queens limit worker size strategically: small workers were no less successful foragers, so that producing a larger number of smaller workers may overall increase queen fitness. We found that queens were less likely to attack large workers, perhaps because attempting to coerce large workers is riskier. However, larger workers did not forage less, did not invest more in ovarian development, and were not more aggressive themselves. There was therefore little evidence overall that queens limit conflict by producing smaller workers.

**Significance statement:**

In social animals, parents might manipulate phenotypic traits of their offspring in their own interests. In paper wasps (*Polistes*), the first offspring produced are smaller than the queen and become workers: instead of founding their own nests, they stay and help their mother to rear new queens and males. We investigated whether *P. gallicus* queens could benefit by producing small daughter workers by using cross-fostering to create size mismatches between queens and their offspring. We then recorded foraging activity, reproductive effort, and aggression on nests. Queens were less likely to attack larger workers, but overall, there was limited evidence of size-based queen–worker conflict. However, because small workers were no less successful foragers, producing a larger number of smaller workers may optimize colony work effort.

## Introduction

The environment that an offspring experiences during its development often has profound effects on its future prospects. In many organisms, critical features of that environment are provided by the mother, and maternal effects should be especially likely when mothers actively provision offspring with resources that influence offspring phenotype. In cooperatively breeding and eusocial taxa, a social group commonly comprises a single breeder or breeding pair together with non-reproductive individuals known as “helpers” or “workers,” which are offspring of the breeders. The reproductive success of breeders is usually strongly correlated with the number of non-reproductives present in the group and with the extent to which they provide help (e.g., Shreeves and Field 2002). There is therefore considerable potential for parents to strategically manipulate offspring phenotype in their own interests (Russell and Lummaa 2009).

In social insects, breeders (queens) are nearly always larger than their worker offspring. Body size in Hymenoptera has been shown to be largely environmentally determined (Kovacs et al. 2010) and correlated with larval diet (Karsai and Hunt 2002; Brand and Chapuisat 2012), and in many social insects, the future queen (foundress) provisions the first worker-destined offspring alone. A queen could therefore actively manipulate attributes of her offspring, such as their body size, by controlling the quantity and quality of resources that she provides during their development (Reinhold 2002; Wolf and Wade 2009; Kapheim et al. 2011; Lawson et al. 2017). Here, we focus on two non-exclusive ways in which future queens could benefit from manipulating offspring size. First, a queen may benefit by producing smaller worker-destined offspring if those offspring are then less able to compete with her. Unless they are genetically identical, the members of a social group are likely to have different evolutionary interests, and family conflicts have been particularly well studied in social insects. Conflicts between the queen and her workers can occur over the sex ratio of offspring, female caste fate, length of queen tenure, worker ovarian development, shares of reproduction, and work effort (Bourke 1994; Cant and Field 2001; Ratnieks et al. 2006). For example, workers may prefer to lay the male eggs produced by the colony and may prefer to work less hard than the queen herself would prefer (Ratnieks and Reeve 1992; Cant and Field 2001). These conflicts of interest suggest that cooperation might be enforced by coercion or the threat of coercion (Ratnieks and Wenseleers 2008; Cant 2011). Body size is a likely proxy for resource-holding potential. Thus, in social species where the queen uses aggression to establish physical dominance over workers and deter them from starting fights (Cant et al. 2006; Jandt et al. 2014), a queen that produces small workers might minimize potential conflict or competition from her offspring, and be better able to physically dominate or coerce them if conflict does occur.

A second possibility is that the queen may manipulate the size of her future workers primarily to optimize total colony foraging success. The quality and quantity of reproductives eventually produced by a colony depend on how much provisioning is performed by the workers. In Hymenoptera, foraging abilities are often positively correlated with body size (reviewed in Bosch and Vicens (2006)). To increase the foraging ability of her workers, a queen might therefore increase their individual size, so that maximizing worker foraging ability might conflict with minimizing queen–worker conflict (see above). However, the size of individual workers is likely to trade-off with the number of workers that the queen is able to produce. In addition, foraging is a risky activity (see Cant and Field (2001) and references therein): a large worker is more costly to produce but might have the same mortality rate as a small worker (or even a higher rate under some conditions: Couvillon and Dornhaus 2010). Thus, even if workers that are twice as large do more than twice as much work while they are alive, after accounting for mortality risk, producing smaller workers in greater numbers could be beneficial for queens.

In our temperate paper wasp (*Polistes*) study system, colony foundresses (future queens) are larger than their workers and are responsible for almost all of the reproduction, even though workers are physiologically able to mate and lay eggs. In this study, we investigate why *P. gallicus* queens limit the size of the workers they produce. Producing large workers might be advantageous in some ways, for example if they are better foragers. But large workers may also be costly; the queen might not so easily dominate them when there is conflict. Queen and worker sizes are naturally positively correlated (see below). This suggests that worker size might be the result of a trade-off: queens may produce optimal-sized workers, as large as possible to maximize foraging efficiency, but not so large that they cannot be controlled when there is conflict. We investigate the hypotheses discussed above by testing whether the size of workers affect the amount and efficiency of their foraging (foraging effort and foraging success), their reproductive effort (ovarian development), and the amount of aggression in the group. One difficulty with investigating these hypotheses using unmanipulated social groups, especially given the observed natural correlation between queen and workers sizes, is to determine how queens would interact with workers that differ in size from their biological workers. Indeed, despite the existence of long-standing hypotheses to explain why workers are smaller than queens (e.g., Alexander 1974), there have been few convincing empirical tests. We take a novel approach by using cross-fostering of queens between nests to experimentally create size mismatches of the kind that could occur during ongoing evolutionary conflict (Kolliker and Richner 2001). We thereby force queens to interact with workers of different sizes, while ensuring a constant queen–worker relatedness of zero. If queens normally optimize worker size in their own interests, perhaps against the interests of the workers themselves, we expect that while cross-fostered workers that are larger than the queen’s biological workers may be better foragers, they will result in negative outcomes for queens through greater realized conflict (greater worker ovarian development or aggression).

## Material and Methods

### Data collection

#### Species/population

The life cycle of *P. gallicus* can be divided into three phases: (1) In the spring, each overwintered foundress (future queen) starts the construction of her paper nest alone. During this founding (pre-worker) phase, she lays eggs in the nest cells and then provisions for the developing larvae herself. This first brood is exclusively female and the adults become workers. (2) During the worker phase, workers carry out most of the foraging and brood care, while the queen continues to lay eggs. (3) During the reproductive phase, reproductive offspring, both male and female, emerge and disperse after mating.

We studied the *P. gallicus* population in and around Medina-Sidonia in Andalusia, Southern Spain, from 19 March to 26 June 2016 and from 11 April to 16 June 2017. Nests were found attached to prickly pear cactus plants (*Opuntia* sp.). During the pre-worker phase, we marked and recorded the location of newly founded nests and surveyed each nest before dawn every other day until the first pupa appeared, and then every day until the end of the experiment. During surveys, we recorded the presence/absence of the queen, the oldest brood stage in the nest, the number of pupae, and the number of workers. To minimize observer bias, blinded methods were used when all behavioral data were recorded and/or analyzed.

#### Cross-fostering

Ten days after the first pupa had appeared on a nest, the nest and its queen were carefully removed temporarily from the substrate. For genetic analyses (see “Genotyping”), we collected three eggs from the nest and stored them in a tube of 100% ethanol. We individually marked queens with three dots of enamel paint on the thorax and measured the length of their right forewings using digital calipers. The wing was measured twice. If the two measurements differed by more than 0.2 mm, they were discarded and the wing was measured again. If the difference was smaller than 0.2 mm, we used the average of the two measurements. The queens were then released where they were collected, but each nest was replaced with a foreign one. Replacements were chosen randomly, although we did not use nests located less than 5 m apart, and we avoided reciprocal swaps unless only two nests were available on a given day. Cross-fostered nests were reattached in the exact location of the original nest and the queen was introduced directly onto the nest. Almost all queens accepted the cross-fostered nest (only 4 out of 102 were absent when we visited the nest the next day).

#### Video recording

Twelve days after the emergence of the first worker on each cross-fostered nest, we collected the workers, measured their right forewings (as described above in “Cross-fostering”), and marked them with one dot of enamel paint on the thorax, using different colors for individuals coming from the same nest. The workers were reintroduced onto their nest 1–2 h later. All workers present during our experiments were already pupae at the time of cross-fostering, and were provisioned by the original queen only.

Two days later (14 days after the first worker emerged), we video recorded activity from 11.30 am to 4.30 pm (the period of peak wasp activity) on all the nests that were still active (*N* = 32). A digital video camera (Sony HDR-CX450, Sony HDR-PJ330, or Panasonic HC-V520) on a tripod was set up approximately 1 m in front of the nest, looking into the cells. The video cameras were covered with a cardboard sunshade to prevent them from overheating.

The day after videoing, we collected the nest and the wasps. All individuals were freeze-killed and then stored in tubes of 100% ethanol. Adults, pupae, and larvae were stored individually, and eggs were kept in groups of five.

#### Genotyping

Before calculating the natural correlation between the size of queens and their biological workers, we genotyped queens and putative biological workers at 16 DNA microsatellite loci (see “Appendix” for protocol). When the queen was not collected, we used the three eggs collected during cross-fostering to partially reconstruct her genotype. Only workers that had genotypes consistent with being the queen’s daughters were included in the analysis. The correlation between queen size and average biological worker size was calculated from a sample of 53 queens and 199 workers.

We also calculated the correlation between queen size and average cross-fostered worker size from 36 queens and 149 workers. We additionally genotyped cross-fostered queens and the workers collected after videoing. We used the genotypes to check the relatedness of cross-fostered queens to the workers on their new nests with the program Relatedness 5.08 (Queller and Goodnight 1989).

### Data analyses

#### Worker mismatch

To test whether a variable was affected by the cross-fostered workers being smaller or larger than the queen’s biological workers, we used “worker mismatch.” Worker mismatch was calculated as the difference in size between an individual cross-fostered worker and the average size of the queen’s biological workers, taking into account the direction of the size difference (a negative worker mismatch indicates cross-fostered workers smaller than biological workers). At the nest level, we used an “average worker mismatch,” the difference between the average size of cross-fostered workers and the average size of the queen’s biological workers. Because the biological workers of most of the cross-fostered queens were not available, the size of biological workers was estimated from the queen-biological worker size correlation (see “Results”). Note that all cross-fostered workers present during our experiments were already pupae at the time of cross-fostering, and so were provisioned entirely by the original queen.

#### Video analyses

We obtained data from 32 cross-fostered *P. gallicus* nests including a total of 117 workers. From the videos taken in the field, we collected data on foraging activity and aggression. We recorded departures from and arrivals to the nest by all wasps and whether a visible resource was brought back to the nest. We calculated foraging effort at the individual level (time a wasp spent off nest foraging) and colony foraging effort at the nest level (the summed amount of time all wasps spent off the nest foraging). We similarly calculated foraging success at the individual level (the number of prey balls brought back to the nest during video-recording by a wasp) and colony foraging success at the nest level (the summed number of prey balls brought back to the nest during video-recording by all the wasps).

We also recorded aggressive interactions between cross-fostered queens and workers (Table 1). We first recorded whether there were aggressions between queens and workers: whether each worker initiated aggression towards the queen during video-recording, and whether the queen initiated aggression towards each worker. For workers where there were aggressions, we then calculated the number of aggressive interactions initiated by the worker and initiated by the queen, for each worker and in total in each nest. In addition, we recorded whether aggressive interactions preceded a worker’s departure from the nest (occurred within 10 s of a worker leaving).

**Table 1:** List of aggressive behaviors recorded

Behavior	Description
Darting	Wasp suddenly moving towards another individual, no physical contact
Chasing	Wasp following another individual, no physical contact
Licking	Wasp licking another individual
Chewing	Wasp chewing on another individual
Mounting	Wasp sitting on top of another individual
Lunging	Darting with physical contact
Grappling	Wasps fighting with their front legs
Stinging	Wasp stinging/trying to sting another individual (including “C” posture)
Harassing	Wasp following another individual, with physical contact

We also recorded data for other variables such as number of workers on the nest, number of larvae or brood in the nest, date, temperature, and sunshine (calculated as the proportion of time the nest was in the sun rather than shade during the video).

#### Ovary dissections

**Fig. 1:**
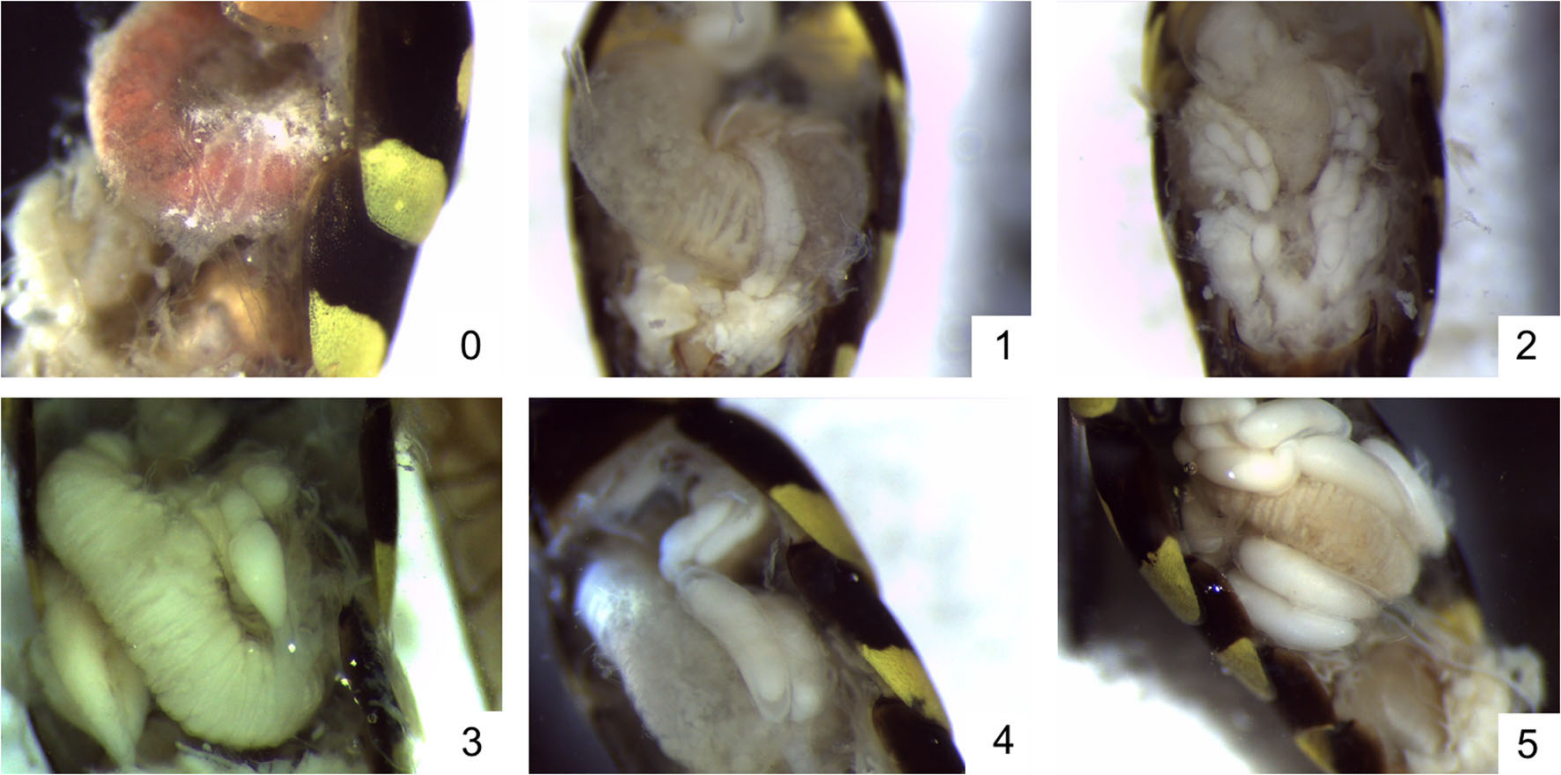
Ovarian development scores: 0 = complete absence of development, the ovariole is translucent; 1 = some slight thickening, the ovariole is opaque in appearance but there is no evidence of chorionated eggs; 2 = small, chorionated egg(s) present; 3 = small-medium egg(s) present; 4 = medium-large egg(s) present; and 5 = large, fully formed egg(s) present

We dissected workers and queens in Ringer’s solution under a Leica M80 microscope. The developmental condition of each of the six ovarioles was scored qualitatively on a scale of 0 to 5 (with zero denoting the complete absence of development and five representing the largest, fully formed eggs; Fig. 1). These data were used to calculate the average ovarian development score for each individual. We also measured the largest egg from each wasp using a Leica MZ6 binocular light microscope. We found a strong positive correlation between the length of the most developed egg and ovarian development (*t* = 25.52, *P* < 0.001, *R*^2^c = 0.85), regardless of the generation of the wasp (worker or queen). Therefore, we use only the ovarian development score in our analyses.

### Statistical analyses

#### Size and foraging

The relationship between the size of a queen and the mean size of her workers was analyzed using Pearson’s correlations.

Analyses of worker behavior (foraging, aggression, and ovarian development) were conducted both at the individual level and at the nest level. At the individual level, worker foraging effort was analyzed using a linear mixed model (Packages lme4 and lmerTest; Bates et al. 2015; Kuznetsova et al. 2017). Conditional *R*-squared values (*R*^2^c) were calculated with the MuMIn package (Barton 2017). The full model included proportion of sunshine, temperature, number of larvae per worker, and worker mismatch as explanatory variables, with date and nest identity as random variables. Foraging success could not be transformed to follow a normal distribution and did not fit any other distributions; it was therefore analyzed using Spearman’s rank correlations. The explanatory variables included in the full model for foraging effort were used individually in Spearman’s rank correlations. At the nest level, whole colony foraging effort and foraging success were analyzed using linear mixed models. Full models included proportion of sunshine, temperature, number of workers, number of larvae, and average worker mismatch as explanatory variables, with date as a random variable.

#### Aggression

At the individual level, whether a the queen initiated aggressions towards a worker and whether a worker initiated aggressions towards the queen were analyzed using generalized linear mixed models with a binomial distribution and logit link function, with worker mismatch and time spent on the nest as explanatory variables, and date and nest identity as random variables. The number of aggressions initiated by the queen towards each worker and the number of aggressions initiated by a worker towards the queen could not be transformed to follow a normal distribution and did not fit any other distributions; they were therefore analyzed using Spearman’s rank correlations and Kruskal–Wallis tests. At the nest level, the number of aggressions initiated by the queen and the number of aggressions initiated by the workers were log-transformed and analyzed using linear mixed models. The variables included in the full models and correlations were the same as for the analysis of foraging (see above).

#### Ovarian development

We compared ovarian development between queens and workers using a linear mixed model and a Kruskal–Wallis test. At the individual level, worker ovarian development did not follow a normal distribution and could not be transformed to do so; Spearman’s rank correlations were therefore used to analyze the data with queen ovarian development and worker mismatch as individual explanatory variables. At the nest level, average worker ovarian development was analyzed using a linear model. The explanatory variables tested were number of brood, foraging effort, number of aggressions, queen ovarian development, and average worker mismatch.

For all models, non-significant variables were manually removed one by one. The final models, presented in the results, are the ones that minimize the AIC. We report P-levels throughout the results for the focal explanatory variable “worker mismatch.” For other covariates, we report P-levels only when they are significant at the *P* < 0.05 level. Similarly, only significant (*P* < 0.05) Pearson’s and Spearman’s rank correlations are presented. All analyses were performed with the statistical software R (R Core Team 2012).

## Results

### Queen-worker size correlation and relatedness

**Fig. 2:**
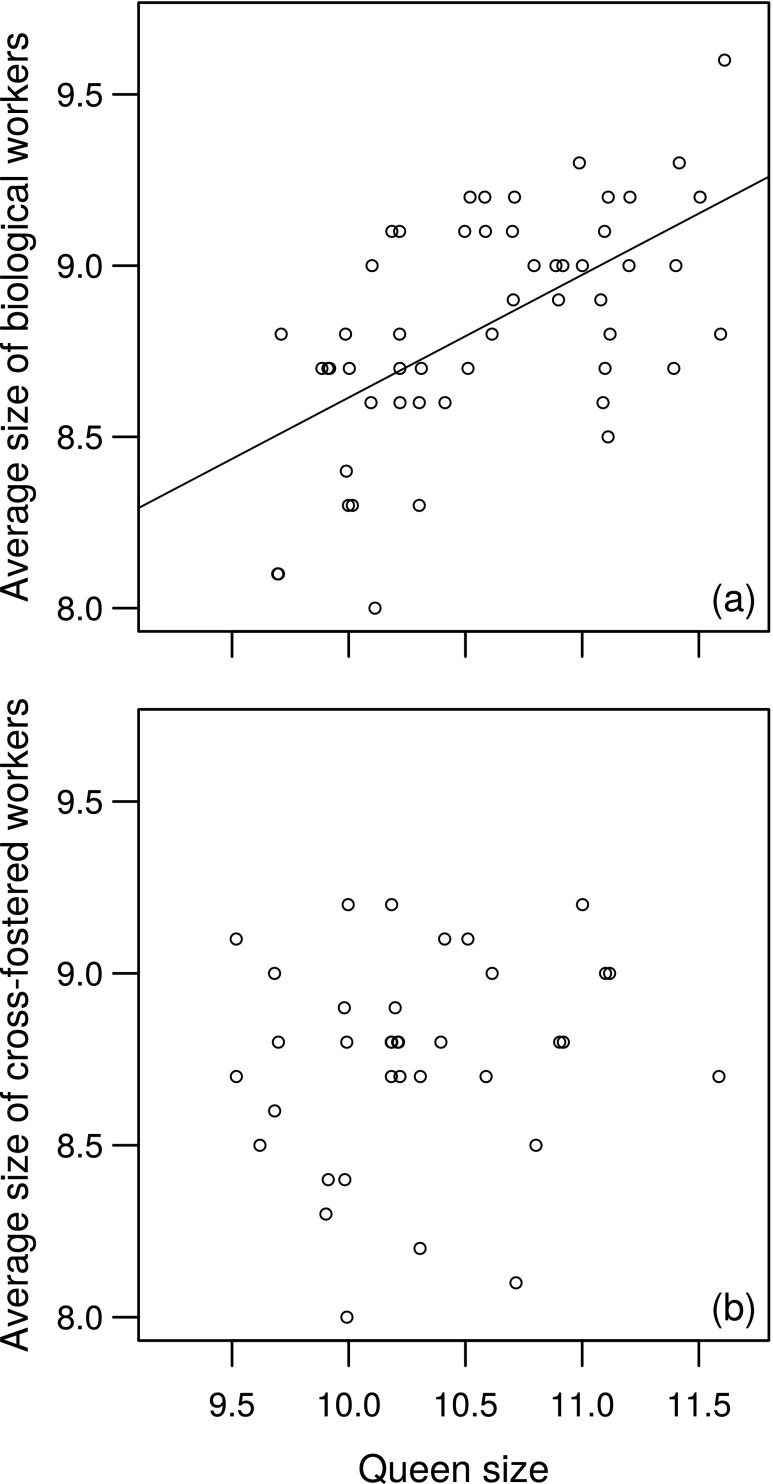
Correlation between the size (mm) of queens and the size of **a** their biological workers or **b** their cross-fostered workers. Line is from least-squares regression

A queen’s size was positively correlated with the average size of her biological workers (*r* = 0.58, *P* < 0.001; Fig. 2a). Workers measured on average 84% of the size of the queen (mean worker size = 8.7 mm, mean queen size = 10.4 mm), and no individual workers were larger than their mothers.

In all cross-fostered nests, workers were smaller than the new queen, both on average (Fig. 2b) and individually. However, cross-fostering successfully created a size mismatch: queen size and the average size of her cross-fostered workers were not correlated (Fig. 2b). Worker mismatch varied from − 1.1 to + 0.8 mm (cross-fostered workers being 91 to 109% of the size of biological workers) at the individual level, and average worker mismatch per nest varied from − 0.9 to + 0.5 mm (91 to 107%) at the nest level. As expected, mean relatedness between cross-fostered queens and the workers on their new nests was only slightly above zero (0.088 ± 0.025). In contrast, relatedness between nest-mate workers, expected to be daughters of the original queen, was 0.73 ± 0.026, close to the value expected for full haplodiploid sisters.

### Foraging

**Fig. 3:**
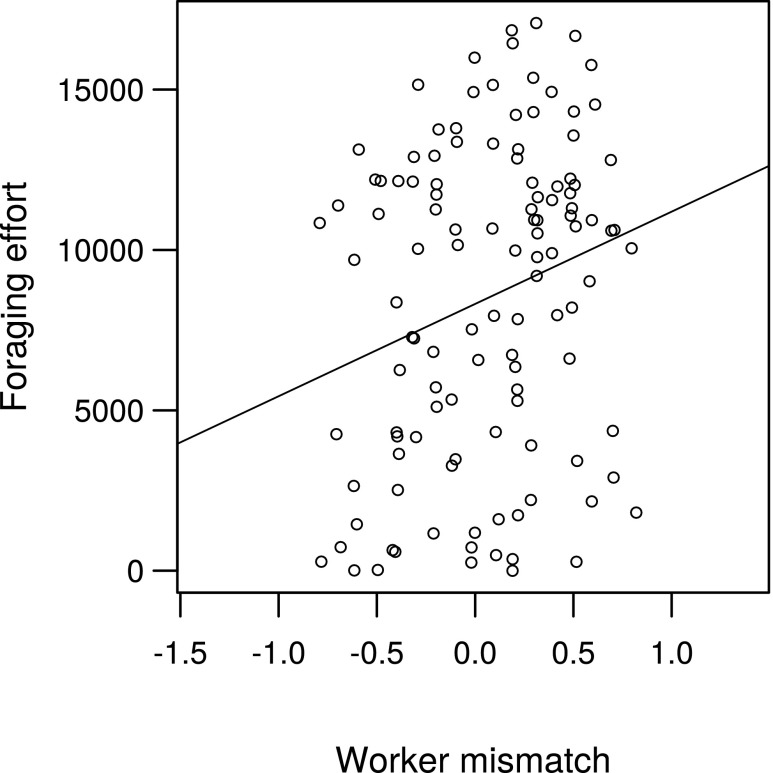
Relationship between worker foraging effort (time spent foraging (s)) and worker mismatch (mm). Line is from least-squares regression

**Fig. 4:**
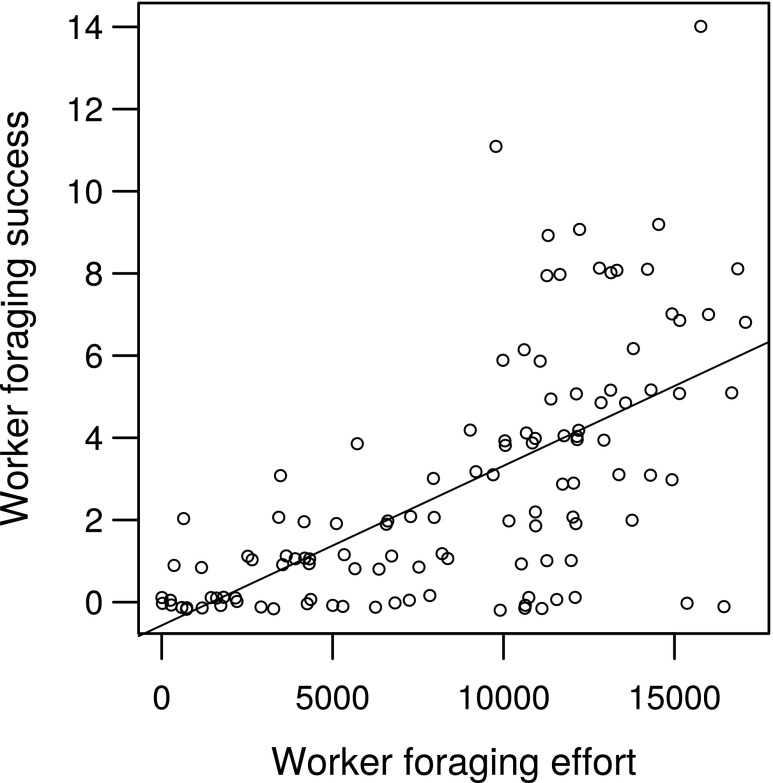
Relationship between worker foraging success (number of prey balls brought back to the nest) and worker foraging effort (time spent foraging (s)). Line is from least-squares regression

At the individual level, worker foraging effort (time a worker spent off nest foraging) increased with worker mismatch (*t* = 2.10, *P* < 0.05, *R*^2^c = 0.15, Fig. 3), as well as with the number of larvae per worker on the nest (*t* = 2.22, *P* < 0.05). Larger workers foraged more but were not more successful; the number of prey balls brought back to the nest (foraging success) was not affected by worker mismatch (rho = 0.15, *P* = 0.10), or any of the other tested variables. Foraging success increased with foraging effort (rho = 0.67, *P* < 0.001; Fig. 4); workers foraging for longer were overall more successful.

At the nest level, foraging effort and foraging success were, unsurprisingly, very strongly correlated with the number of workers present on the nest (respectively *r* = 0.85, *P* < 0.001, and *r* = 0.60, *P* < 0.001); the more workers present, the more time collectively spent foraging and the more prey balls brought back to the nest. We therefore used average time spent foraging per worker and average foraging success per worker as measures of nest foraging effort and nest foraging success. We found that neither nest foraging effort nor nest foraging success was affected by average worker mismatch (respectively *t* = −0.43, *P* = 0.67, *R*^2^c = 0.13, and *t* = 0.50, *P* = 0.62, *R*^2^c = 0.32). Foraging success at the nest level was positively correlated with the number of larvae per worker present on the nest (*t* = 2.21, *P* < 0.05, *R*^2^c = 0.32).

### Aggression

**Fig. 5:**
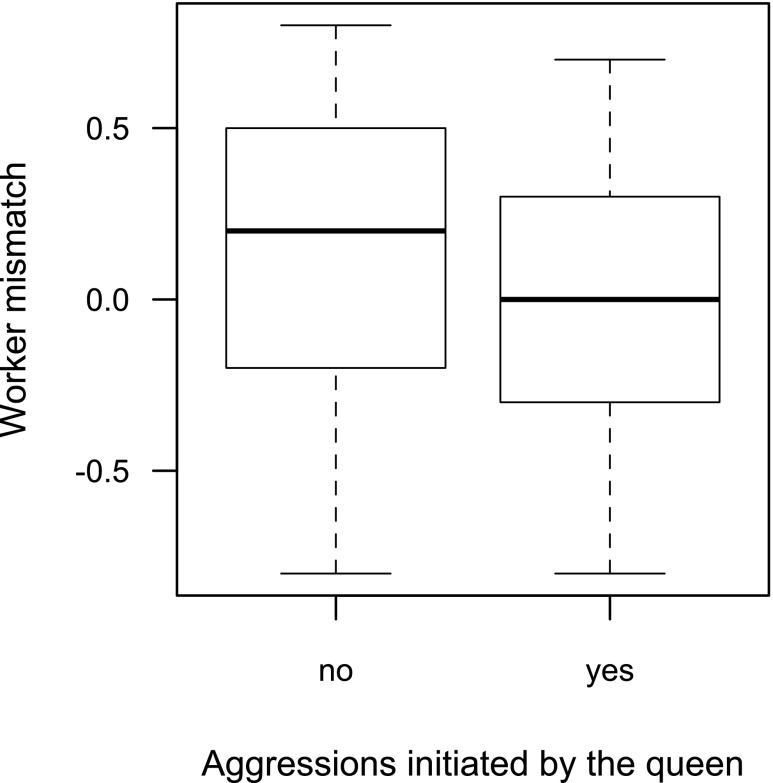
Worker mismatch (mm) according to whether or not a worker received aggression from the queen

Aggressive interactions were recorded between queens and 65% of the workers present in the video recordings. At the individual level, worker mismatch (*z* = − 496, *P* < 0.001, *R*^2^c = 0.40) but not time spent on the nest, was correlated with whether the queen initiated aggressions towards a worker or not: workers that received at least one aggression from the queen were on average smaller (Fig. 5). The number of aggressions initiated by the queen towards the workers that she did attack was not affected by worker mismatch (rho = − 0.02, *P* = 0.86). Whether a worker initiated aggressions towards the queen was not affected by worker mismatch (*z* = − 1.54, *P* = 0.12, *R*^2^c = 0.34) nor was the number of aggressions initiated by the workers that did attack the queen (rho = 0.10, *P* = 0.47).

At the nest level, average worker mismatch (and all of the other tested variables) had no significant effect on whether the queen or the workers initiated aggressions (respectively *z* = − 0.04, *P* = 0.97, *R*^2^c = 0.09, and *z* = − 0.09, *P* = 0.93, *R*^2^c = 0.10), or on the number of aggressions initiated by the queen and the number of aggressions initiated by the workers if aggressions did occur (respectively *t* = − 0.09, *P* = 0.93, *R*^2^c = 0.07, and *t* = 0.30, *P* = 0.78, *R*^2^c = 0.15).

Queens did not generally use aggression to prompt worker foraging. Out of the 1561 worker nest departures, only 2.6% were preceded by an aggression (within 10 s of the worker leaving), and only around half of these aggressions (23/40) were from the queen as opposed to other workers.

### Ovarian development

**Fig. 6:**
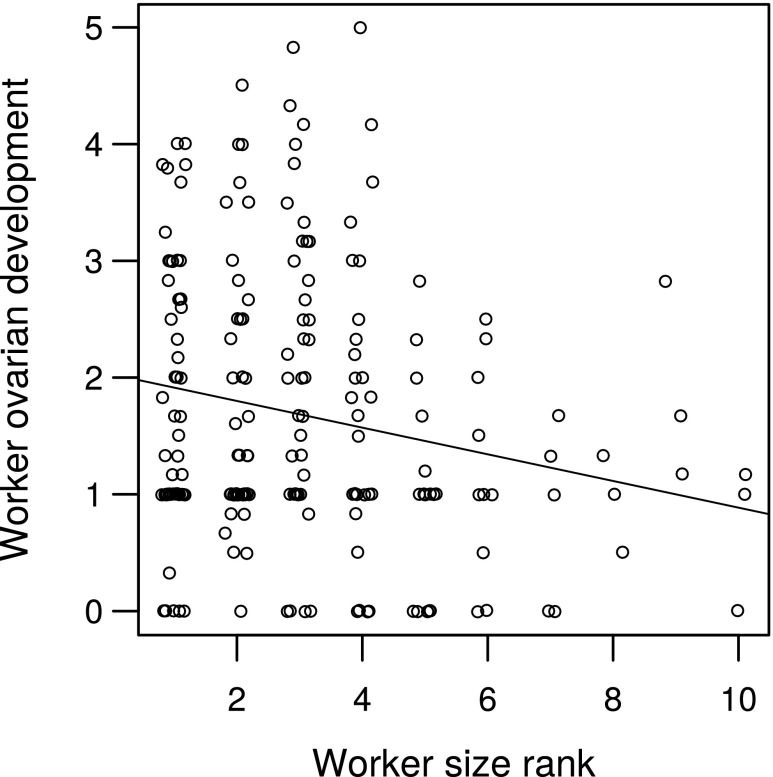
Relationship between worker ovarian development score and worker size relative to other workers in the nest (size rank = 1 for the largest worker in a nest). Line is from least-squares regression

As expected, workers had less-developed ovaries than queens (Kruskal–Wallis *χ*^2^ = 65.003, *df* = 1, *P* < 0.001). At the individual level, worker mismatch was not correlated with worker ovarian development (rho = 0.10; *P* = 0.14); larger workers did not have more developed ovaries. However, size rank, the relative size of a worker compared to that of the other workers in the nest, was correlated with ovarian development. The largest workers in a nest had the most developed ovaries (rho = − 0.17; *P* < 0.05; Fig. 6). Note that the correlation is negative because the largest worker in a nest is denoted rank 1.

At the nest level, worker mismatch had no effect on worker ovarian development (*t* = − 1.33, *P* = 0.20; GLM: *F*_(2, 29)_ = 3.93, *P* = 0.03, *R*^2^ = 0.16). However, workers had more developed ovaries in nests where the number of brood was smaller (*t* = − 1.94, *P* < 0.05).

## Discussion

In the paper wasp *P. gallicus*, we used cross-fostering to create size mismatches between queens and workers against a background of almost zero relatedness. We found that worker size relative to the size of the queen’s biological workers (“worker mismatch”) was correlated with worker foraging effort: relatively larger workers foraged more. In addition, relatively larger workers were less likely to receive aggression from the queen. These effects were detectable at the individual level, but not at the nest level, perhaps because of less variation in body size at the nest level, and because our sample size at the nest level was smaller. While worker mismatch was correlated with foraging effort and aggression, it was not correlated with foraging success or ovarian development. We note that cross-fostered workers that are larger than biological workers are more likely to be larger individuals per se, and vice versa. Worker mismatch and absolute worker size were indeed positively correlated (rho = 0.87, *P* < 0.001 at the individual level, and rho = 0.83, *P* < 0.001 at the nest level), so that effects of worker mismatch could also reflect the absolute size of workers. We now discuss our findings with special reference to the hypotheses outlined in the introduction, that queens might benefit by limiting worker size if this reduces conflict/competition and/or optimizes colony foraging effort.

One of the hypotheses we considered, to explain why queens might produce relatively small workers, was in order to optimize total colony foraging success. Our results provide some support for this hypothesis. In contrast with bumblebees, in which larger workers have a higher foraging success than smaller workers (Goulson et al. 2002; Spaethe and Weidenmuller 2002), larger *P. gallicus* workers were not more successful foragers. At the individual level, foraging success simply increased with foraging effort; the more time a worker spent off nest foraging, the more prey balls she brought back. We also found that foraging success at the nest level increased with the number of workers present. The more workers present, the more time spent foraging in total, and the more prey balls brought back to the nest overall. Therefore, in order to maximize nest foraging success, a queen might produce as many workers as possible. However, since the amount of provisioning a queen can perform is limited, she faces a size-number trade-off: producing small workers should allow her to produce more of them.

Foraging success, at both the individual and the nest levels, increased with the number of larvae per worker in the nest of *P. gallicus*. As suggested in *Polybia occidentalis* where foraging rates match the number of larvae present in the nest (Howard and Jeanne 2005), and *Polistes dominula* where workers appear to adjust their helping effort according to offspring need (Donaldson et al. 2014), foraging activity in *P. gallicus* seems to be demand-driven.

A second hypothesis we considered was that a queen strategically produces small workers that are less able to compete with her. A queen may be less able to dominate and coerce larger workers, so that conflict of interests over workload (such as foraging effort) or ovarian development might increase. We found little consistent evidence for this. Queens were less likely to be aggressive towards relatively larger workers, which might reflect queens being less willing to risk attempting to coerce better-matched individuals. However, worker mismatch did not affect aggression in the opposite direction, by workers themselves towards the queen. Larger workers also did not invest more than smaller workers in ovarian development and did not forage less.

In taxa where workers have the potential to reproduce directly, for example by surviving to inherit the egg-laying position, there is a potential conflict of interest over worker foraging effort, with queens preferring workers to work harder than their own optimum (Cant and Field 2001). Foraging is costly in terms of energy expenditure and predation risk, and mortality rates of foraging workers are higher than for workers on the nest (Wilson 1985; Cant and Field 2001). If queens have to force workers to leave the nest and forage, producing small workers that are easier to coerce into foraging might be advantageous. However, we found that although larger *P. gallicus* workers spent more time off nest foraging than smaller ones, as observed in bumblebees and ants (Goulson et al. 2002; Kelber et al. 2010), queens did not appear to directly coerce workers into leaving the nest to forage. We found that very few nest departures (1.5%) were likely to have been prompted by aggression from the queen. She might, however, stimulate worker foraging via other means; for example, by indirectly increasing worker activity in general, as perhaps occurs in *Polistes fuscatus* (Reeve and Gamboa 1987). Alternatively, she might control reproduction but not worker foraging, as in *Polistes versicolor* and *Ropalidia marginata* (Bruyndonckx et al. 2006; De Souza and Prezoto 2012).

In social insects, there is often a trade-off between individual reproductive effort and work effort (foraging, nursing larvae etc.); workers invest energy in developing their ovaries at the expense of work (Hillesheim et al. 1989; Martin et al. 2002; Wenseleers et al. 2003). In addition, larger workers may have more developed ovaries or more viable oocytes (e.g., Sullivan and Strassmann 1984; Blacher et al. 2017). Selection might then favor queens that produce smaller workers that invest less in their own reproduction, and that devote more energy to colony work. In fact, we found that worker ovarian development was not affected by worker mismatch. There was, however, an effect of the size of a worker relative to that of the other workers in the nest: the largest workers in a nest had the most developed ovaries. This could reflect a size-based dominance hierarchy among the workers where low-ranked workers perform risky tasks such as foraging (Reeve 1991), while high-ranked workers stay on the nest and are more likely to reproduce. In bumblebees, for example, dominant workers inhibit ovarian development of other workers (Bloch and Hefetz 1999).

We also found that worker ovarian development was greater in nests with a smaller number of brood. In bumblebees, workers increased their ovarian development in response to the absence of brood (Sibbald and Plowright 2014). In *P. dominula*, workers appear to use brood abundance as a cue for queen quality and fertility, increasing their ovarian development and egg-laying in response to a decrease in brood numbers (Liebig et al. 2005). Therefore, our result could reflect an increased investment in worker ovarian development when the queen is less able to assume laying responsibilities herself. Alternatively, smaller numbers of brood could be a consequence, rather than a cause, of worker ovarian development. If the queen has to spend time policing workers and selectively destroying worker-laid eggs (Ratnieks 1988; Ratnieks and Wenseleers 2008), she might have less time and/or energy available to lay her own eggs.

In social insects, size is often a factor in the outcomes of agonistic interactions (Hughes and Strassmann 1988; Heinze and Oberstadt 1999), and experience in aggressive contests often affects behavior; individuals that lose contests tend to avoid engaging in aggressive interactions (Hsu et al. 2006). We therefore expect larger workers, which are closer in size to the queen and could possibly win in a contest, to be more aggressive towards the queen than smaller workers, and queens to preferentially be aggressive towards smaller workers that they are able to easily physically dominate. We found that although the queen was more likely to be aggressive towards smaller workers, larger workers were not more aggressive themselves: they were as likely as smaller workers to be aggressive towards the queen; and if they were aggressive, they attacked the queen a comparable number of times.

As well as the hypotheses we have focused on here, there are other potential explanations for differences between queen and worker body sizes. One long-standing explanation is that by restricting them nutritionally during their development, queens effectively manipulate offspring into becoming workers in the first place (Alexander 1974; Charnov 1978; Crespi and Ragsdale 2000; Kapheim et al. 2015; Lawson et al. 2016). All else being equal, for the queen, there is a clear fitness benefit from producing reproductive offspring with the help of her daughters (queen’s relatedness to her offspring = 0.5), rather than allowing her daughters to reproduce independently (queen’s relatedness to her grandoffspring = 0.25) (Hamilton 1964; Charnov 1978). Smaller individuals may be less able to survive and reproduce on their own, and might therefore benefit by staying on their mother’s nest, rather than taking the risk of attempting to reproduce alone (Ratnieks and Wenseleers 2008). This has been suggested as a mechanism for the evolution of eusociality across a variety of taxa, although there are few convincing demonstrations of its importance, perhaps because it is difficult to test directly (Alexander 1974; Charnov 1978; Queller 1996; Crespi and Ragsdale 2000; Kapheim et al. 2015).

Other explanations for differences between queen and worker body sizes do not imply queen manipulation. For example, differences might be passive effects of seasonal changes in resource availability and/or quality (Mousseau and Dingle 1991; Karell et al. 2008). In temperate regions, resources may be scarcer or less nutritious at the beginning of the season, when the queen is provisioning her first (worker) offspring, than later in the season when next year’s reproductives (new queens and males) are provisioned (Poethke et al. 2016). In some ants and termites, workers from the first offspring brood (“nanitic” workers) are indeed smaller than subsequent workers. Nanitic workers may result from the queen being able to provide only a limited amount of food compared to when there are lots of workers to tend the brood (Light 1943; Porter and Tschinkel 1986; Heinze et al. 2003). Similarly, in primitively eusocial insects, including *Polistes*, total foraging effort is greater towards the end of the nesting season when new queens are developing, since workers are by then providing for the offspring, increasing the adult/larva ratio (Kamm 1974; Reeve 1991). The fact that workers do not need to overwinter, and therefore may not require large energy reserves, could also contribute to the size difference, although workers are also smaller than queens in many tropical species where there is no winter (Jeanne and Fagen 1974; Noll et al. 1997).

In summary, we used cross-fostering to create size mismatches between queens and workers of the paper wasp *P. gallicus.* We found that worker mismatch was positively correlated with foraging effort and negatively correlated with whether aggressions were initiated by the queen. However, worker mismatch had no effect on worker foraging success or ovarian development. The size mismatches we could induce reflect the range of worker sizes naturally available. In future studies, larger mismatches could be induced by manipulating worker size beyond the natural range. Worker size might be experimentally decreased by starving larvae during the founding phase, and increased by providing queens with prey ad libitum (Mead and Pratte 2002) or by hand-feeding larvae directly (Karsai and Hunt 2002). In addition, future studies could investigate other potential effects of worker size and relatedness, such as effects on mortality, and could measure reproductive success directly over a longer time period (e.g., Leadbeater et al. 2011).

## Data Availability

The datasets generated during and/or analyzed during the current study are available from the corresponding author on reasonable request.

## Appendix

### Genetic analyses

DNA from adult legs and whole eggs was extracted using a hotshot extraction method (Truett et al. 2000). DNA from larvae was extracted using an ammonium acetate method (Nicholls et al. 2000). Previously published microsatellite markers for *P. dominula* (Pdom) (Henshaw 2000), *P. chinensis antennalis* (Pc) (Tsuchida et al. 2003; Saigo and Tsuchida 2010), and *Polistes bellicosus* (Pbe) (Strassmann et al. 1997) were found to successfully amplify *P. gallicus* loci. Loci were labeled using dyes from the DS-33 (Applied Biosystems) dye set and divided into two multiplex reactions. Loci were amplified using Qiagen multiplex PCR kit (Qiagen, Venlo, The Netherlands) with the following reaction: 10–100 ng template DNA, 3 μl of 2 × Multiplex master mix (3 mM MgCl_2_) and 1 μl of primer mix with a drop of mineral oil added to prevent evaporation. The primer mix for Multiplex 1 consisted of 0.326 μmol each of Pdom1 and Pdom20, and 0.186 μmol each of Pbe128TAG, Pc63, Pc68, Pdom2, Pdom7, Pdom25, and Pdom140. Multiplex 2 consisted of 0.466 μmol of Pbe430; 0.326 μmol each of Pbe411, Pdom139, and Pc76; and 0.186 μmol each of Pc02, Pc41, and Pc83. PCR were performed on a G-storm GS2 thermal cycler. Multiplex reaction one followed a temperature profile of 95 °C for 15 min; 35 cycles of 94 °C for 30 s, 57 °C for 90 s and 72 °C for 60 s, followed by a final extension step of 60 °C for 30 min. Multiplex 2 followed an identical profile but with a lower annealing temperature of 52 °C. PCR products from larvae and adults were diluted 64-fold while those from eggs were diluted 8-fold. The diluted products were then run on a capillary 3730 Sequencer with Genescan LIZ 500 size standard. Alleles were called by eye using GeneMapper 3.7 (Applied Biosystems).
